# Rational Immunogenicity Modulation of Virus-Like Particles by VLPIM

**DOI:** 10.34133/csbj.0063

**Published:** 2026-04-28

**Authors:** Chufan Wang, Yeyun Chen, Ming-Wei Wang, Lei Ren, Qingtong Zhou

**Affiliations:** ^1^ Research Center for Medicinal Structural Biology, National Research Center for Translational Medicine at Shanghai, State Key Laboratory of Medical Genomics, Ruijin Hospital, Shanghai Jiao Tong University School of Medicine, Shanghai 200025, China.; ^2^ Institute of Artificial Intelligence, Xiamen University, Xiamen 361005, China.; ^3^ Shanghai Innovation Institute, Shanghai 200234, China.; ^4^ Research Center for Deepsea Bioresources, Sanya, Hainan 572025, China.; ^5^ Department of Pharmacology, School of Basic Medical Sciences, Fudan University, Shanghai 200032, China.; ^6^ Department of Chemistry, School of Science, The University of Tokyo, Tokyo 113-0033, Japan.; ^7^ Engineering Research Center of Tropical Medicine Innovation and Transformation of Ministry of Education, School of Pharmacy, Hainan Medical University, Haikou 570228, China.; ^8^ Key Laboratory of Biomedical Engineering of Fujian Province University/Research Center of Biomedical Engineering of Xiamen, Department of Biomaterials, College of Materials, Xiamen University, Xiamen 361005, China.; ^9^ State Key Lab of Physical Chemistry of Solid Surfaces, Xiamen University, Xiamen 361005, China.

## Abstract

Virus-like particles (VLPs) are versatile platforms for drug delivery and vaccine development, but precisely customizing their immune profiles for specific clinical applications remains challenging. Here, we present a computational framework that integrates complementary artificial-intelligence-driven tools for protein design, structure prediction, and antigenicity assessment to enable rational immunogenicity modulation of VLPs while preserving their structural integrity. Leveraging the central role of CD4^+^ T cells in adaptive immunity, this framework first combines class II major histocompatibility complex (MHC-II)-restricted epitope prediction with structure-guided sequence optimization to generate a focused mutation library. Subsequently, its virtual screening workflow combines immunogenicity predictions and structural modeling to pinpoint variants likely to attenuate or enhance CD4^+^ T cell activation while preserving VLP integrity. This framework enables a broader and more efficient exploration of the mutation space to identify VLP structural variants with tailored immunogenicity profiles; hence, we term it the VLPIM (Virus-Like Particle Immunogenicity Modulation) platform. Using hepatitis B core VLPs as proof of concept, VLPIM generated a mutation library of 120 variants, computationally filtered down to 4 candidates, and ultimately identified 2 variants that retained native virus-like morphology, as confirmed by transmission electron microscopy. In vitro immunogenicity assays confirmed that epitope substitutions could either suppress or enhance CD4^+^ T cell activation. This generalizable pipeline permits precise immunogenicity modulation of VLPs and other multimers, accelerating their development as targeted drug carriers and next-generation vaccine platforms. VLPIM is available as both a locally executable tool on GitHub (https://github.com/RuijinHospitalVNAR/VLPIM) and an online web server (https://ruijinhospitalvnar.github.io/Toolboxes/VLPIM_Web_services/).

## Highlights

• **Background:** Virus-like particles (VLPs) are versatile platforms for drug delivery and vaccine development, but precisely customizing their immune profiles for specific clinical applications remains challenging.
• **Main advances:** Leveraging the vital role of CD4⁺ T cells in adaptive immunity, we applied rational design and artificial-intelligence-guided virtual screening (Virus-Like Particle Immunogenicity Modulation [VLPIM]) to pinpoint variants likely to regulate CD4⁺ T cell activation while preserving VLP integrity.
• **Applications:** Using the native hepatitis B virus capsid-derived VLP (HBc VLP) sequence and characteristics as inputs, VLPIM generated VLP variants that preserved the native virus-like morphology, while in vitro immunogenicity assays confirmed that epitope substitutions could either suppress or enhance CD4⁺ T cell activation.
• **Conclusion:** VLPIM permits precise immunogenicity modulation of VLPs and other multimers, facilitating their development as targeted drug carriers and next-generation vaccine platforms.

## Graphical Abstract

**Figure:**
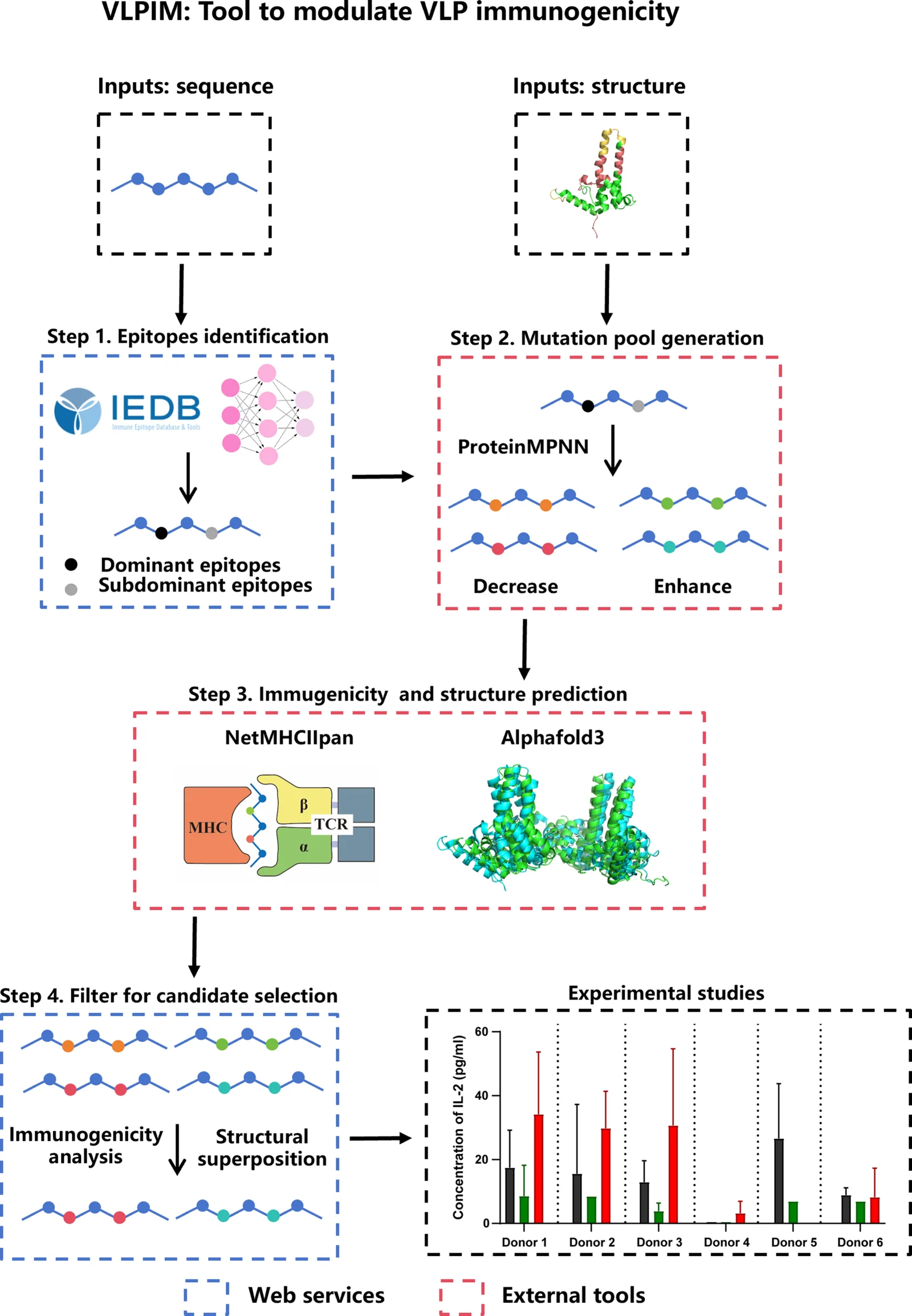

## Introduction

Virus-like particles (VLPs) are a class of unique nanoscale carriers in drug delivery and vaccine design owing to their structural and sequence features [1,2]. These noninfectious particles are inherently safe, exhibit highly ordered virus-mimetic architectures, possess well-defined sites for functionalization, and contain enclosed cavities suitable for molecular encapsulation [3]. Such characteristics enable VLPs to efficiently load and deliver diverse cargos, ranging from small molecules to macromolecules [4]. In addition, their surface topology enables multivalent antigen display, which enhances antigenicity [5,6]. As a result, VLPs hold substantial promise for biomedical applications. Despite these advantages, the intrinsic immunogenicity of VLPs poses challenges for clinical translation. In vaccine applications, the absence of viral genomic material means that VLPs often lack intrinsic adjuvanticity, which may compromise the robustness and durability of immune responses [7,8]. Beyond vaccine development, VLPs are increasingly used as nanocarriers for drug delivery and targeted therapeutics, where carrier-directed immune responses can accelerate clearance and compromise efficacy [9,10]. When VLPs display weakly immunogenic or self-derived antigens, immunodominant responses against structural proteins may suppress the desired response through antigenic competition [11]. These challenges are compounded by preexisting immunity to prevalent viral capsids such as hepatitis B virus, which can trigger rapid recall responses upon administration [12]. Collectively, these considerations underscore the importance of developing strategies to precisely regulate the immunogenicity of VLPs, thereby maximizing their potential as nanocarriers and immunization platforms.

CD4^+^ T cells serve as a central nexus between innate and adaptive immunity, orchestrating effective responses to pathogens and tissue damage. Following antigen capture and processing, antigen-presenting cells display peptide fragments on class II major histocompatibility complex (MHC-II), which are recognized by T cell receptors on CD4^+^ T cells [13,14]. This interaction triggers downstream signaling cascades that promote cytokine secretion, costimulatory molecule expression, and the activation of B cells and effector T cells, ultimately ensuring robust primary responses and durable immunological memory [15,16].

Strategies such as targeted deletion or substitution of epitopes have demonstrated potential in mitigating undesired immune activation [17,18]. However, these approaches are often labor-intensive and time-consuming, and the systematic reconfiguration of multiple antigenic sites while preserving protein structural integrity remains an unresolved challenge. Recent advances in deep learning have facilitated the development of powerful models capable of accurately predicting immunogenicity, such as NetMHCIIpan and DeepMHCII, thereby complementing experimental methods that are limited in their ability to interrogate large mutational landscapes [19–22]. To date, most applications have centered on therapeutic proteins, particularly antibody humanization, with the primary aim of reducing immunogenicity [23–25]. These approaches typically rely on increasing sequence similarity to naturally occurring human proteins through humanization strategies or sequence-based scoring methods [26,27]. However, VLPs and many nonhuman protein therapeutics lack a corresponding human sequence space that can guide humanization-based approaches. Consequently, immunogenicity modulation in such systems generally relies on identifying and modifying predicted MHC-II-restricted CD4^+^ T cell epitopes [28]. Importantly, most existing computational deimmunization frameworks have been developed for monomeric proteins, where mutations can be primarily evaluated on the basis of local sequence or structural constraints [29]. In contrast, VLPs are multimeric assemblies whose structural integrity depends on cooperative interactions between multiple capsid subunits. Mutations introduced to reduce predicted T cell epitopes may therefore compromise particle assembly, capsid stability, or quaternary structure if multimeric constraints are not explicitly considered [30]. A key enabling factor for addressing this challenge is the recent improvement in protein structure prediction accuracy for multimeric complexes, particularly with the development of AlphaFold3 (AF3) [31].

To address this gap, we developed Virus-Like Particle Immunogenicity Modulation (VLPIM), a computational platform designed to modulate the immunogenicity of VLPs (Figs. 1 and 2) and thus expand their potential in biopharmaceutical development. After identifying dominant epitopes via the Immune Epitope Database (IEDB) [32] and/or predicting peptide–MHC-II binding affinities through NetMHCIIpan [33], a comprehensive mutation library was generated using the inverse-folding model ProteinMPNN [34] (Fig. 1). Candidate sequences were then systematically evaluated through an integrated workflow combining NetMHCIIpan for immunogenicity prediction and AF3 for high-accuracy structural modeling. This approach enables the selection of variants with altered immunogenicity while preserving their structural integrity. Application of this framework to hepatitis B core VLPs (HBc VLPs) demonstrated that multiple in silico designed variants retained native virus-like morphology and exhibited distinct immunogenicity profiles. VLPIM distinguishes itself through 4 key innovations: (a) a multimer-aware structural filtering strategy that explicitly considers intersubunit interactions during mutation evaluation; (b) a bidirectional design mode that enables both immunogenicity reduction and enhancement; (c) an integrated workflow combining NetMHCIIpan, ProteinMPNN, and AF3 for comprehensive mutation screening; and (d) an accessible web-based platform that automates the entire pipeline for users without computational expertise.

**Fig. 1.**
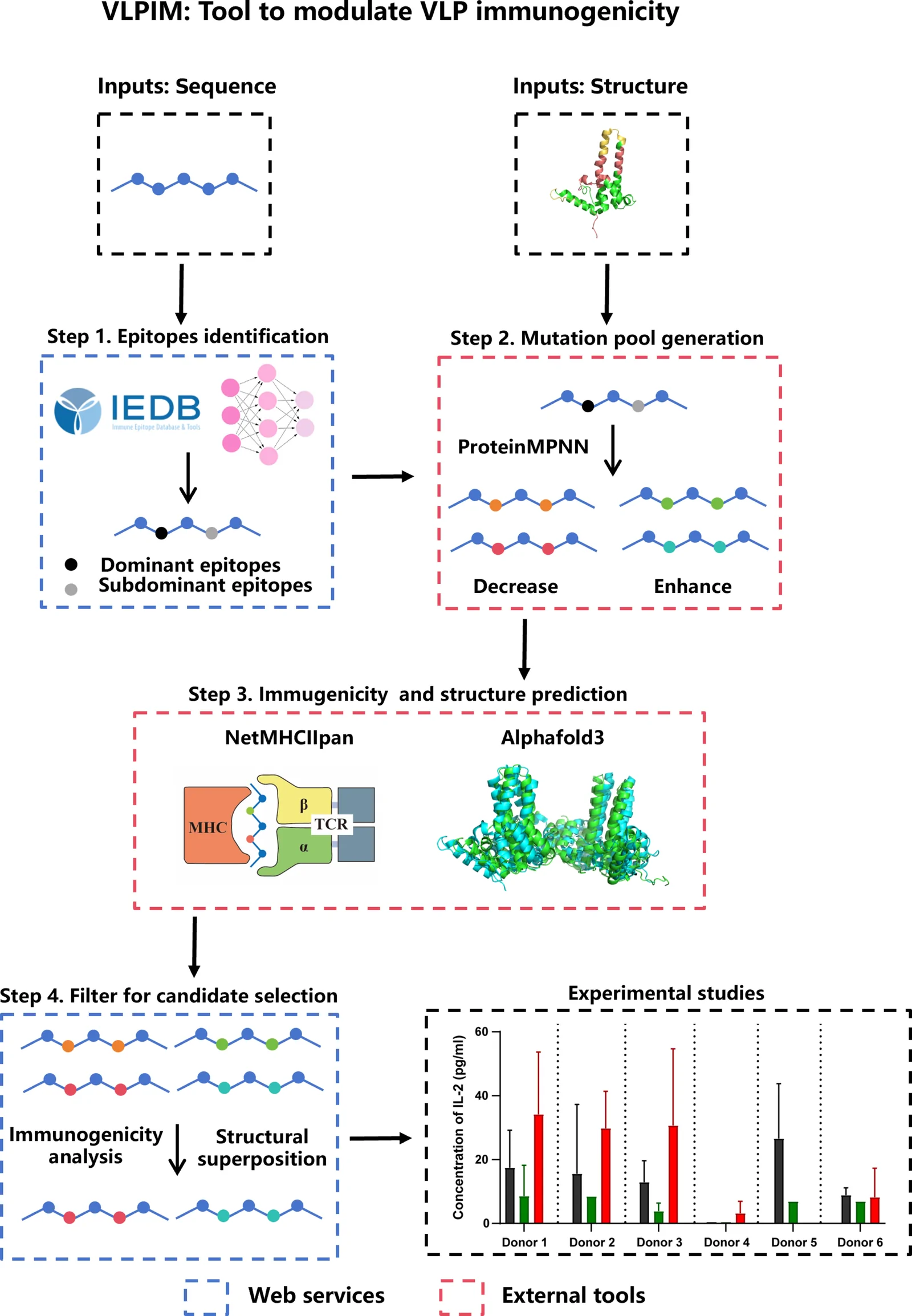
Workflow of Virus-Like Particle Immunogenicity Modulation (VLPIM). Step 1: Dominant and subdominant antigenic epitopes are first identified using Immune Epitope Databases and the NetMHCIIpan prediction model. Step 2: These epitopes provide the basis for generating a mutant protein library through ProteinMPNN-based deep learning methods. Step 3: Structural and immunogenicity predictions are then applied to evaluate the effects of mutations. Step 4: Candidate variants are further filtered using AlphaFold3 (AF3) outputs, Cα root mean square deviation (RMSD), and Rosetta. Finally, the most promising mutants are validated in experimental studies to assess their physicochemical properties and immunogenicity. To distinguish the integrated platform from external tools, the workflow diagram outlines steps processed by the VLPIM web server with blue dashed boxes and those relying on external tools (e.g., AF3 and NetMHCIIpan) with red dashed boxes, although all steps can be executed locally.

**Fig. 2.**
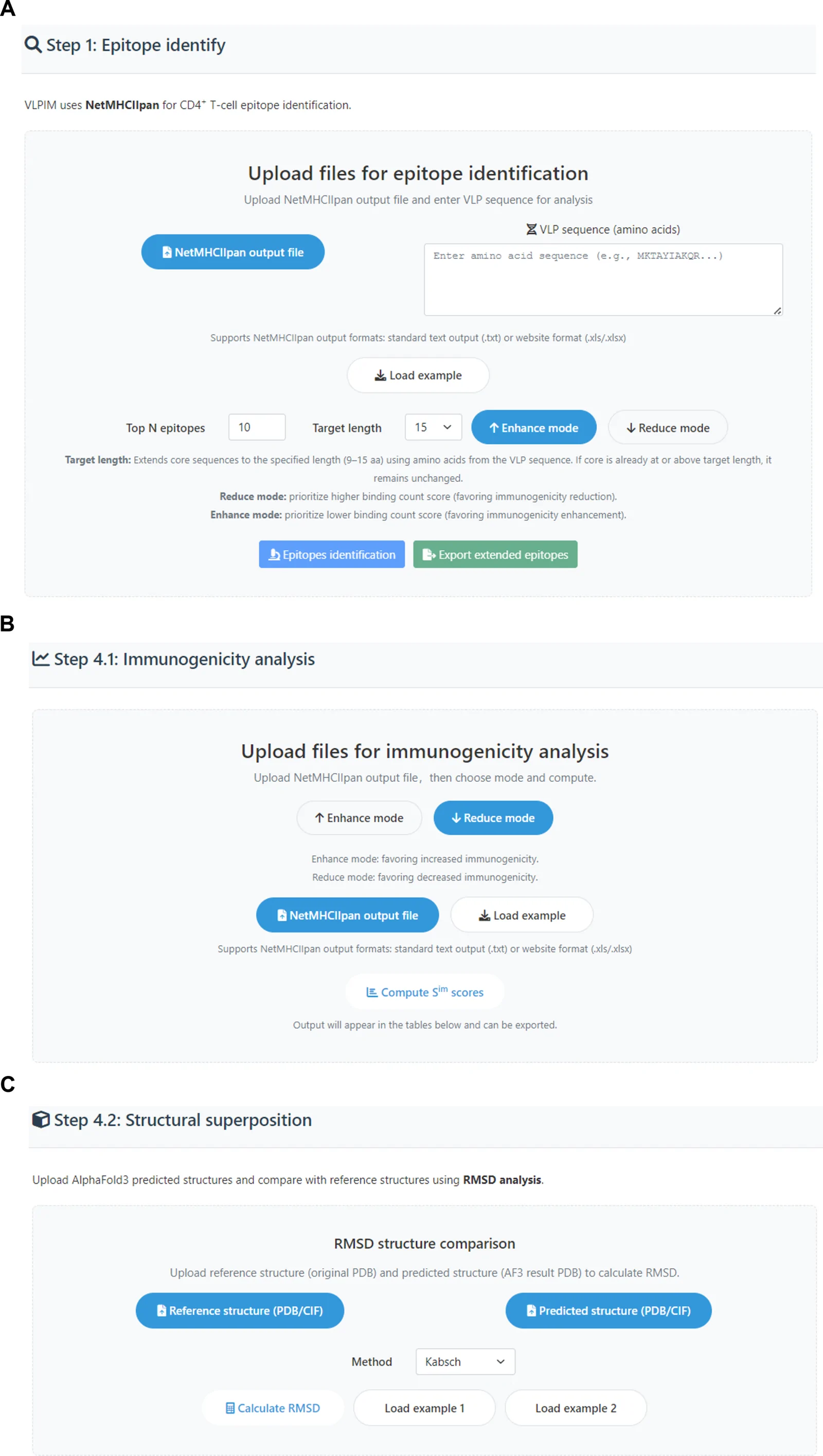
User interface of the Virus-Like Particle Immunogenicity Modulation (VLPIM) web server. (A) Epitope identification module: Users upload the target protein sequence along with NetMHCIIpan prediction results. This module allows customization of key parameters, including design mode, target epitope length, and the number of epitopes displayed. (B) Immunogenicity analysis module: Users provide NetMHCIIpan predictions derived from the generated mutation pool/library and choose the appropriate mode for assessing immunogenicity changes. (C) Structural comparison module: Users upload the reference structure and AlphaFold3 (AF3)-predicted mutant structures. The calculated root mean square deviation (RMSD) values assist in filtering and identifying of optimal candidates for experimental validation.

By making VLPIM freely accessible as a web server (https://ruijinhospitalvnar.github.io/Toolboxes/VLPIM_Web_services/) (Fig. 2) and a standalone tool that can be downloaded and executed locally from GitHub (https://github.com/RuijinHospitalVNAR/VLPIM), we aim to empower the scientific community with a user-friendly tool for VLP immunogenicity modulation, thereby advancing the clinical translation of VLP-based therapeutics and vaccines.

## Materials and Methods

### Implementation of VLPIM web server

The VLPIM web server (Fig. 2) was developed using Vue.js (v2.6.14) [35], a reactive JavaScript framework [36] that enables dynamic data binding and responsive user interactions, and deployed on GitHub pages. The user interface layout and styling were handled through Bootstrap 5.1.3 [37] to ensure a consistent and adaptive design across devices. Font Awesome 6.0.0 [38] was integrated to provide a standardized icon set for enhanced usability and visualization. To support computational and data management functions, 2 JavaScript libraries were incorporated. SheetJS (XLSX) enables users to import and export data tables, including sequence lists and scoring results, in Excel-compatible formats. SVD-JS was utilized to perform singular value decomposition and coordinate alignment, allowing real-time root mean square deviation (RMSD) calculations and structural comparison to be conducted directly within the browser.

The VLPIM web server supports VLP sequence inputs of up to approximately 1,000 amino acids, although shorter sequences (<500 residues) are recommended for optimal performance. Structural inputs are optimized for Protein Data Bank (PDB) files containing up to 500 residues per chain. NetMHCIIpan output files should follow the standard format, and very large files (>10 MB) may require extended processing time. User-configurable parameters are constrained within predefined ranges to ensure stable execution. These include the number of epitopes to select (1 to 50; default, 10), epitope length (9 to 15 amino acids; default, 15), RMSD cutoff values (0.5 to 500 Å; suggested range, 2 to 300 Å), and RMSD iteration cycles (1 to 20; default, 5). In terms of computational performance, epitope identification typically completes within 5 to 30 s, depending on the sequence length and the number of epitopes requested, while RMSD calculations generally require 10 to 60 s, with longer runtimes expected when higher iteration counts are applied. External application programming interface (API) calls are subject to a 10-s timeout. All computations are performed locally within the user’s browser without server-side data storage, we recommend using adequate browser memory to analyze large datasets.

### Local installation, execution, and output of VLPIM

VLPIM supports flexible input formats and provides a user-friendly pipeline for immunogenicity modulation of VLPs (https://github.com/RuijinHospitalVNAR/VLPIM). The protein sequence file must be provided in FASTA format to ensure compatibility with AF3 and NetMHCIIpan. For structural topology input, .pdb or .cif formats are recommended.

1. Installation. The VLPIM script can be installed easily via Python’s package manager, pip, by executing the following command: pip install VLPIM. VLPIM requires Python 3.9 or higher. During installation, users should ensure that all dependencies are correctly installed for full functionality of integrated tools such as ProteinMPNN, NetMHCIIpan, and AF3.
2. Execution. Once installed, VLPIM can be executed from the command line by specifying the required input parameters: python immunogenicity_optimization_pipeline.py --fasta protein.fasta --pdb protein.pdb --mode reduce. In this example, the script takes a protein sequence file (protein.fasta) and a corresponding structural file (protein.pdb) and performs immunogenicity reduction (--mode reduce). Additional optional parameters can be included to refine design settings, output paths, and computational behavior.
3. Help and parameter configuration. Users can view the full list of configurable parameters by running: VLPIM --help. This will display detailed descriptions of all available command options, including paths to sequence (--fasta), epitopes file (--epitopes), and structure (--pdb) files; design mode selection (--mode reduce/enhance); mutation generation settings for ProteinMPNN (--temperatures, -- samples-per-temp); AF3 structure prediction limits (--max-candidates); and output control (--output_file). This built-in help interface allows users to flexibly adapt the pipeline for different design objectives.
4. Output form. After execution, VLPIM generates a comprehensive output file containing designed mutation sequences, scoring metrics, and AF3-predicted structural models of candidate variants. By default, the results are saved to a file named VLPIM_output.txt along with a folder containing predicted structures. Users may customize the output filename using the --output_file parameter as needed.

### Expression and purification of VLP mutants

Mutant expression plasmids carrying ampicillin resistance were synthesized commercially (Shanghai Generay Biotech Co. Ltd., Shanghai, China). Lipopolysaccharide-deficient *Escherichia coli* ClearColi BL21(DE3, LGC Biosearch Technologies, Petaluma, CA, USA) was used as the host. After transformation with plasmid DNA via heat shock, selected colonies were cultured to optical density at 600 nm ≈ 0.6 to 0.8 and induced with 0.1 mM isopropyl-β-d-thiogalactopyranoside at 20 °C for 24 h. Cells were harvested (4,000 rpm for 15 min), resuspended in high-salt lysis buffer, and disrupted by ultrasonication (500 W, 20 kHz, 5-s on/5-s off). Soluble protein was clarified by centrifugation (8,000 rpm for 10 min), subjected to ammonium sulfate precipitation (1:1.5 volume ratio). Pellets were resuspended in 20 mM Tris–HCl (pH 8.0; 0.9% NaCl). Then, these proteins were purified sequentially via size exclusion chromatography (Sepharose CL-4B, GE Healthcare, Chicago, USA) and ion exchange chromatography (DEAE Sepharose Fast Flow, GE Healthcare, Chicago, USA). Final proteins were filtered (0.22 μm) and stored at −20 °C.

### Morphological characterization

The molecular weights of the purified proteins were analyzed by 15% sodium dodecyl sulfate–polyacrylamide gel electrophoresis (SDS-PAGE; Mini-PROTEAN system with PowerPac Basic, Bio-Rad, Hercules, CA, USA). The morphology and particle size of HBc mutants were characterized using transmission electron microscopy (TEM; Tecnai G2 F20 S-TWIN, FEI, Hillsboro, OR, USA).

### Thermal stability analysis

#### Differential scanning fluorimetry

SYPRO Orange (1:500 in phosphate-buffered saline) was used. Protein solutions (25 μM) were mixed with dye (4:1, v/v) and subjected to a temperature gradient (25 to 95 °C, 1 °C/min) in a quantitative real-time polymerase chain reaction instrument (QuantStudio 1 Plus, Thermo Fisher Scientific, Waltham, MA, USA). Experiments were performed in 3 independent replicates.

#### Differential scanning calorimetry

Differential scanning calorimetry (DSC) (VP-DSC, Malvern Panalytical, Malvern, UK) measurements were performed on protein solutions (2 mg/ml; 400 μl) from 25 to 95 °C at 240 °C/h. Baseline-corrected thermograms were analyzed in Origin, and thermal transition midpoint (Tm) values were determined from deconvoluted peaks. A representative DSC thermogram is shown [39]. The obtained Tm values were consistent with the stability trend observed in differential scanning fluorimetry (DSF) assays, supporting reproducibility of this thermal stability assessment.

### Immunogenicity assessment

Human peripheral blood mononuclear cells (PBMCs) were obtained from commercial suppliers, where all samples were collected under institutional-review-board-approved protocols with informed consent. PBMCs were isolated from heparinized human blood via density gradient centrifugation and resuspended in RPMI 1640 medium supplemented with 10% fetal bovine serum (cell viability > 95%). PBMCs (5 × 10^5^ cells per well) were seeded in 96-well plates and stimulated with mutant or wild-type (WT) VLPs (10 μg/ml) for 60 h at 37 °C in a humidified atmosphere containing 5% CO_2_. Supernatants were subsequently collected, and interleukin-2 (IL-2) secretion was quantified by enzyme-linked immunosorbent assay (ELISA) kit (Thermo Fisher Scientific). PBMCs were obtained from 6 independent human donors, which were considered biological replicates. For each donor, ELISA measurements were performed with 4 technical replicates per condition.

## Results

### A rational framework for immunogenicity modulation of VLPs

To optimize VLPs through targeted modulation of CD4^+^ T cell epitopes while preserving overall structural integrity, our computational design framework integrates 4 key computational tools: NetMHCIIpan-4.3 (https://services.healthtech.dtu.dk/services/NetMHCIIpan-4.3/), ProteinMPNN (https://github.com/dauparas/ProteinMPNN), AF3 (https://github.com/google-deepmind/alphafold3), and Rosetta (https://rosettacommons.org/) [40]. NetMHCIIpan enables accurate, high-throughput prediction of immune responses across multiple MHC-II alleles, allowing rapid identification of dominant epitopes. Once these epitopes are confirmed, ProteinMPNN, an inverse folding model, generates possible mutations based on the structure of the designed VLPs. The structural effects of these mutations are then evaluated using AF3, which provides state-of-the-art protein structure predictions. Promising variants identified from these models were further assessed using Rosetta to analyze interface contacts, enabling the selection of candidates with preserved structural integrity. This integrated workflow (Fig. 1) follows 4 main steps: (a) epitope identification, (b) mutation pool generation, (c) immunogenicity and structural prediction, and (d) filter design and candidate selection for experimental validation.

1. Epitope identification. Epitope identification is the first and essential step in the VLPIM workflow. This stage involves preparing the input sequences and parameters needed for downstream analysis. The viral capsid protein sequences are used as the starting point and are queried against the IEDB to collect the peptides with experimentally validated high binding affinity to MHC-II molecules. However, the available database records are often limited in number and may lack detailed quantitative information, making it difficult to pinpoint the most promising mutation sites. To overcome this limitation, we apply the NetMHCIIpan model to predict peptide–MHC-II binding affinities across a wide range of allelic subtypes. By processing these predictions through the epitope identification module of VLPIM, a more comprehensive landscape of potentially mutable epitopes is obtained. In addition to the input files, VLPIM supports several essential parameters for user-defined configurations:

• mode: VLPIM supports 2 design modes: immunogenicity reduction (reduce mode) and immunogenicity enhancement (enhance mode), allowing users to modulate antigenic properties according to specific application needs.
• Target_length: Predicted epitopes from computational tools often differ from experimentally validated epitopes and typically highlight only a 9-amino-acid core epitope. Users can define the full mutation window surrounding the core epitope, typically extending from 9 to 15 amino acids to cover the entire antigenic region.
• epitope_number: To prevent overlapping mutation regions, the number of epitopes selected is constrained by sequence length. For VLP sequences shorter than 200 amino acids, the default maximum number of epitopes is set to 10.

2. Mutation pool generation. After confirming the dominant epitopes, the next step in VLPIM is sequence regeneration using ProteinMPNN. This process requires 2 essential inputs: (a) the candidate sequence and (b) the structural model of the VLP. If no experimentally resolved structure is available, a reliable structural model can be generated using the AF3 online server (https://alphafoldserver.com/). ProteinMPNN allows users to customize key parameters, including the sampling temperature (commonly set at 0.1, 0.2, or 0.3) and the number of sequences to generate (ranging from 20 to higher values depending on the design scope).
3. Immunogenicity and structural prediction. The mutant sequences generated by ProteinMPNN are reevaluated using NetMHCIIpan and AF3. The resulting structural and immunogenicity predictions are subsequently integrated to identify promising mutant candidates for further analysis. For optimal performance, VLPIM recommends that users employ 8 of the most prevalent human MHC-II alleles to ensure broad population coverage: HLA-DRB1*01:01, HLA-DRB1*03:01, HLA-DRB1*04:01, HLA-DRB1*07:01, HLA-DRB1*08:01, HLA-DRB1*11:01, HLA-DRB1*13:01, and HLA-DRB1*15:01 [41,42].
4. Filter design and candidate selection for experimental validation. Following the initial generation of candidate mutant sequences, designs underwent sequential evaluation, including immunogenicity prediction, multimer structural alignment, and energy-based stability assessment. Immunogenicity was quantified using the *S^im^* score, structural consistency of multimer assemblies was evaluated by RMSD, and interfacial stability was assessed using Rosetta-derived energy metrics. Variants were subsequently ranked on the basis of these parameters, and the top candidates were selected for experimental validation.

To enable robust comparison among closely related sequence variants, we represented predicted binding affinities from NetMHCIIpan using median inhibitory concentration (IC_50_) values rather than percentile ranks. Percentile ranks are derived through internal normalization against background peptide distributions and are primarily intended for classification of strong versus weak binders. In contrast, IC_50_ values preserve a continuous affinity scale, allowing finer discrimination among variants with modest differences in predicted binding. Thus, we developed a scoring function based on NetMHCIIpan prediction:

Siim=∑i100−xi−xminxmax−xmin×100if mode=reduce∑ixi−xminxmax−xmin×100if mode=enhance (1)

Here, *S^im^* score calculation uses a rank-based normalization approach. For each human leukocyte antigen (HLA) allele *i*, let *x_i_* represent the rank of a candidate mutant sequence based on its predicted IC_50_ binding affinity among all candidates in the mutation library, where lower IC_50_ values (indicating stronger binding) receive lower rank values. IC_50_ ranks are then normalized using min–max normalization. The final *S^im^* score is calculated by aggregating normalized scores across all alleles, with mode-specific adjustments: In reduce mode, scores are inverted prior to summation to favor weaker binding; in enhance mode, scores are summed directly to prioritize stronger binding. In both modes, a lower *S^im^* score indicates greater immunogenicity modulation, providing a standardized metric for prioritizing candidate mutations. Following immunogenicity analysis, mutant sequences are ranked by their *S^im^* scores, and the top 10 candidates are subjected to structural prediction using AF3.

The structural models generated by AF3 are first superimposed onto the original input structure, with the Cα RMSD value serving as the primary criterion to assess structural similarity, which was used for ranking rather than as a strict exclusion cutoff. RMSD was calculated over backbone α-carbon atoms using an iterative alignment algorithm: An initial alignment was obtained via the Kabsch algorithm, followed by up to 5 cycles of outlier pruning where residues with distance greater than 2.0 Å were removed and the remaining inliers were realigned. The final RMSD was computed on the inlier residues after convergence. Based on the combined ranking, the top 3 mutants were selected for further analysis. Additional key Rosetta-derived interface parameters are considered to refine candidate selection, including (a) Gibbs free energy change per solvent-accessible surface area (dG/dSASA), which reflects binding free energy relative to solvent accessibility; (b) packing quality of the interface (Packstat), which measures packing quality; and (c) buried unsatisfied hydrogen bonds (BUNS), which evaluates unsatisfied hydrogen bonds. These structural and energy-based metrics were used to analyze structural quality and energetic trends among the shortlisted designs and to provide a mechanistic basis for interpreting the subsequent experimental results. The most promising candidates are then advanced for validation by experimental studies.

To facilitate broad adoption, we implemented VLPIM in both a locally executable version and a web-based interface. We implemented the VLPIM web server via GitHub pages [43] (https://ruijinhospitalvnar.github.io/Toolboxes/VLPIM_Web_services/), which integrates essential computational modules for epitope identification (Fig. 2A), immunogenicity analysis (Fig. 2B), and structural superposition (Fig. 2C). The availability of both a standalone, locally executable tool and web server enables users to choose between convenience and full control: The web interface offers ease of use, while local execution ensures sensitive data remain in-house and allows complete parameter customization.

### In silico design of HBc VLPs using VLPIM

HBc VLPs, derived from the hepatitis B virus core protein, are widely used in drug delivery and vaccine development. Therefore, fine-tuning the intrinsic immunogenicity of HBc VLPs is of particular importance. To achieve immune modulation using the VLPIM strategy, we first queried the IEDB to retrieve experimentally validated high-affinity peptides binding to MHC-II molecules. A total of 23 eligible epitope sequences were retrieved. To facilitate subsequent experimental validation and practical applications, we optimized the HBc sequence (UniProt ID: P03146) by removing the C-terminal nucleotide-binding region (residues 150 to 183), thereby minimizing the influence of innate immune response region. After this adjustment, the number of valid epitopes available for statistical analysis was reduced to 20. Among them, the core sequence “WIRTPPAYR” appeared most frequently, accounting for approximately 20% of the total, suggesting that it may represent one of the dominant MHC-II-restricted epitopes and thus a promising target for future mutational studies (Fig. 3A).

**Fig. 3.**
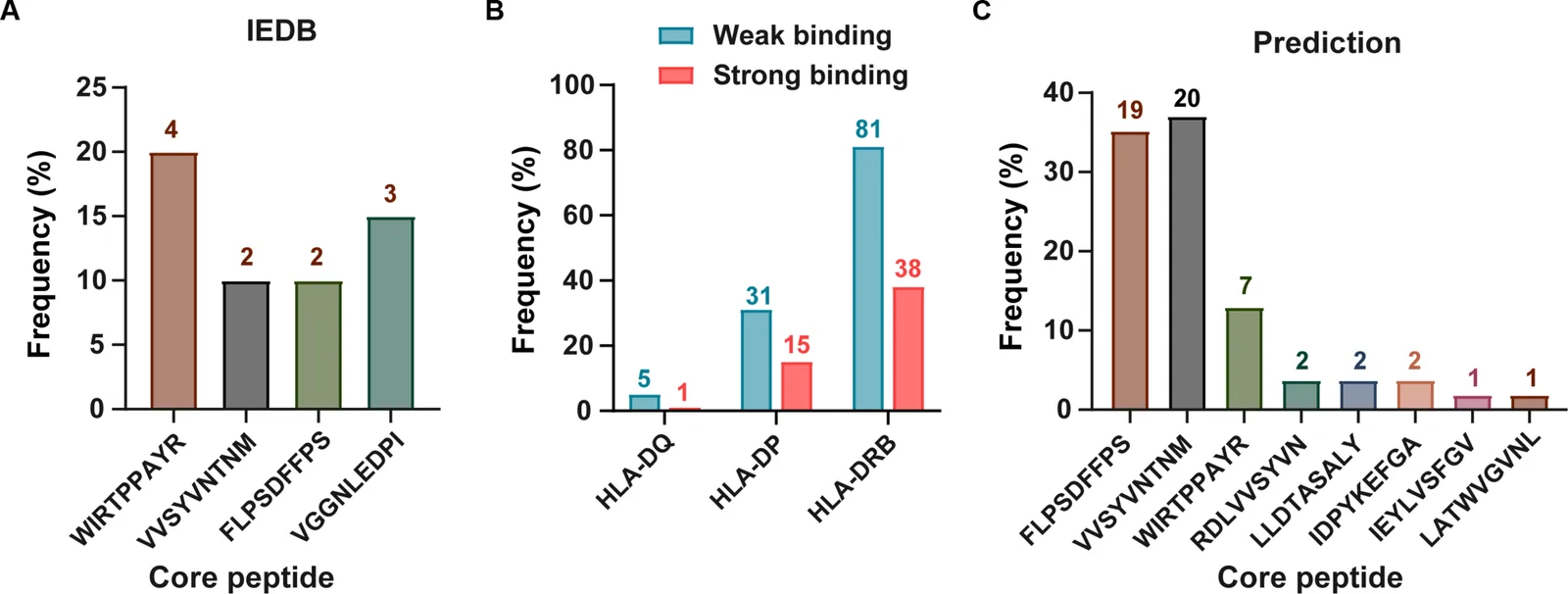
Analysis of class II major histocompatibility complex (MHC-II) antigenic epitope characteristics derived from Immune Epitope Database (IEDB) statistics and NetMHCIIpan predictions, including (A) distribution of experimentally validated antigenic epitopes and (B) allelic variant-specific epitope distribution across MHC-II molecules. Strong binding peptides are defined as percentile rank ≤ 0.500; weak binding peptides are defined as 0.500 < percentile rank ≤ 2.000. (C) Frequency of 9-mer core peptide-binding regions classified as strong binders within predicted MHC-II epitopes, as evaluated by NetMHCIIpan. HLA, human leukocyte antigen.

However, because the available database records were limited in number and often lacked detailed quantitative information, it was difficult to determine the most promising mutation sites solely from existing resources. To address this, we used the NetMHCIIpan model to predict binding affinities between candidate peptides and a broad range of MHC-II allelic subtypes. The predictions (Fig. 3B) revealed that HLA-DRB alleles exhibited the highest number of positive binding peptides, consistent with previous reports [44], and provided important guidance for our subsequent immunogenicity analysis. Furthermore, based on these predictions (Fig. 3C), we identified 2 additional epitopes of interest: FLPSDFFPS and VVSYVNTNM. Notably, the selection of these epitopes is supported not only by computational modeling but also by prior studies, which have confirmed VVSYVNTNM [45] as a commonly recognized MHC-II-restricted epitope.

In this study, 2 hotspot regions were selected for targeted sequence optimization using ProteinMPNN. To preserve structural integrity, we fixed residues outside the selected regions, while we introduced sequence variation within the epitope regions. Within this framework, 2 complementary design strategies were implemented: ProteinMPNN-based deimmunization (PMD), aimed at reducing predicted MHC-II binding affinity, and ProteinMPNN-based immunogenicity enhancement (PMI), aimed at increasing predicted binding affinity (Table 1 and Fig. S2). ProteinMPNN was then used to generate mutant sequences by fixing amino acids adjacent to the PMD or PMI sites. To explore mutation diversity, we applied 3 sampling temperatures (0.1, 0.2, and 0.4) in ProteinMPNN, yielding a total of 60 candidate mutant sequences derived from the resolved structure of HBc VLPs (PDB ID: 6HTX).

**Table 1.** Mutation regions for deimmunization (PMD) and immuno-enhancement (PMI).

Name	Motif	Position
**PMD**	LSFLPSDFFPSVRDL	16−30
	DLVVSYVNTNMGLKF	83−97
**PMI**	DIDPYKEFGATVELL	2−16
	EALESPEHCSPHHTA	40−54

NetMHCIIpan predictions revealed distinct trends in MHC-II binding affinity among the mutated antigen sequences. For the PMD series, the optimal mutant of LSFLPSDFFPSVRDL (MAX MUT) exhibited a pronounced decrease in antigen affinity, approximately 19-fold lower than the WT, while the optimal mutant of DLVVSYVNTNMGLKF (MAX MUT) showed a modest increase, corresponding to an overall ~10-fold reduction in MHC-II binding (Fig. 4A). In contrast, the PMI mutants displayed markedly enhanced binding, with optimal mutants showed ~33- and ~52-fold increases in MHC-II affinity, respectively (Fig. 4B). To maintain overall structural stability, we introduced mutations synchronously into both targeted regions for each series. Therefore, when evaluating candidate sequences, the immunogenicity changes from both epitope regions were summed to obtain the overall score for ranking. Using Eq. 1, the immunogenicity scores for each sequence were calculated and summed for the pairs, and the top 20 sequences with the largest overall shifts in immunogenicity were selected for subsequent structural analysis (Table S1).

**Fig. 4.**
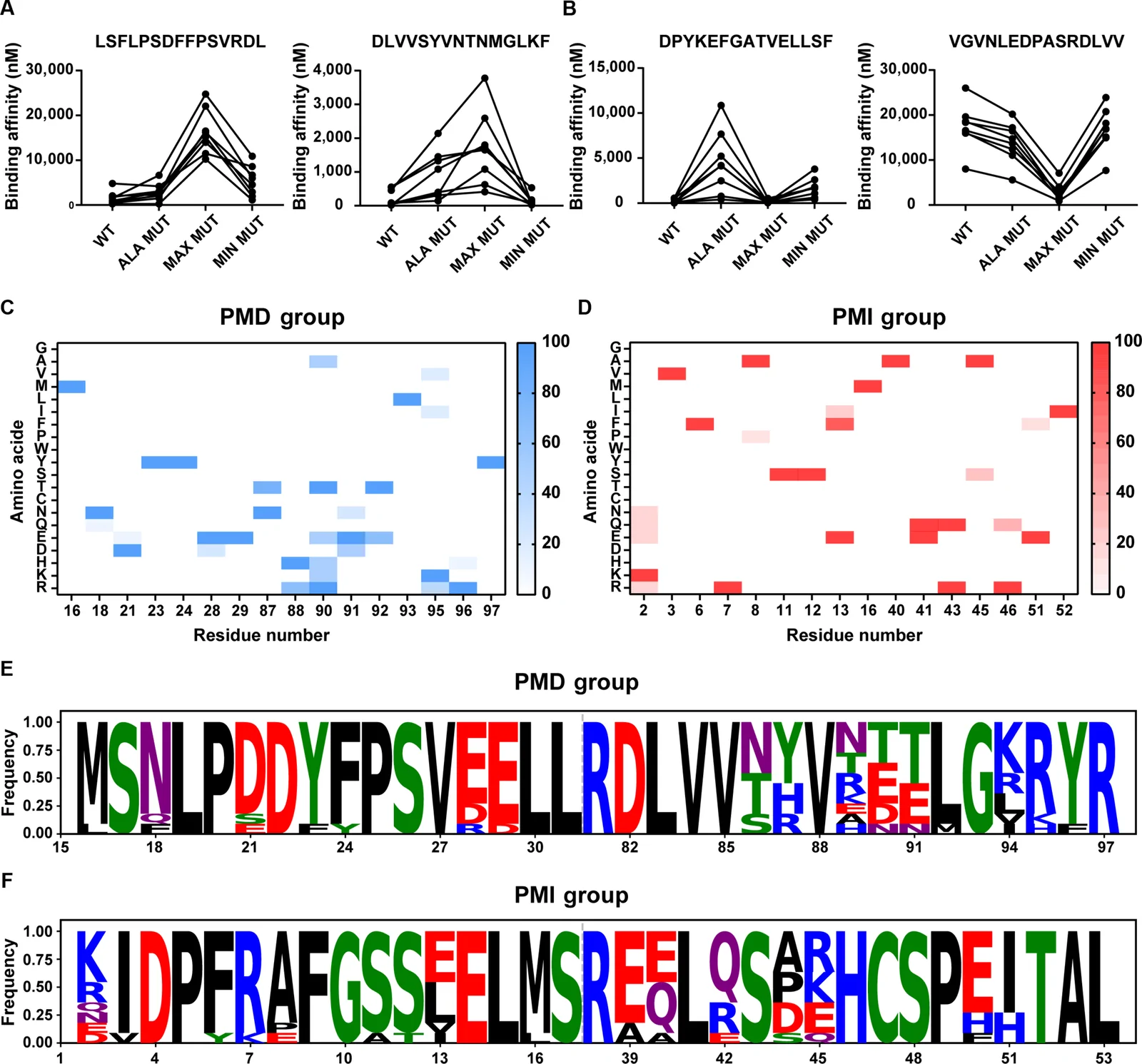
In silico immunogenicity and amino acid preference profiling of deimmunizing and immuno-enhancing hepatitis B core virus-like particle (HBc VLP) mutants designed by Virus-Like Particle Immunogenicity Modulation (VLPIM). (A and B) Changes in predicted median inhibitory concentration (IC_50_) values for antigen–class II major histocompatibility complex (MHC-II) binding affinity in ProteinMPNN-based deimmunization (PMD) (A) and ProteinMPNN-based immunogenicity enhancement (PMI) (B) mutants, as predicted by the NetMHCIIpan model. (C and D) Heatmap analysis of amino acid substitutions in the PMD (C) and PMI (D) mutant series (top 20 variants ranked by *S^im^* score), highlighting deimmunizing and immuno-enhancing mutations, respectively. ALA MUT, alanine mutation; WT, wild-type; MAX MUT, the mutant variant with maximum change in predicted immunogenicity; MIN MUT, the mutant variant with minimum increase (or greatest reduction) in antigen–MHC-II binding affinity for PMD (A) and PMI (B) minimum change in predicted immunogenicity. In heatmaps, different colors distinguish PMD and PMI, and color intensity indicates the magnitude of normalized amino acid mutation frequency. (E and F) Sequence logo analysis comparing amino acid usage frequencies in mutation regions between PMD (E) and PMI (F) mutants. The height of each letter represents the frequency of the corresponding amino acid at that position, revealing distinct amino acid preference patterns between deimmunization and immunogenicity enhancement designs.

To understand the relationship between immunogenicity changes and amino acid preferences in ProteinMPNN-generated mutant sequences, we analyzed the top 20 sequences ranked by predicted antigen affinity changes. For the PMD series, which underwent deimmunization mutations, the amino acid preferences exhibited clear patterns (Fig. 4C). Mutations tended to introduce hydrophilic residues rather than hydrophobic ones, reflecting the binding characteristics of these peptides with MHC-II, where hydrophilic residues are less favorable for forming stable interaction interfaces. In addition, many sites displayed a preference for acidic or basic amino acids, which may introduce electrostatic repulsion within the MHC-II binding groove and further destabilize peptide binding [14]. Similarly, we analyzed the amino acid preferences of the PMI series, which were designed for immunoenhancing mutations. As shown in Fig. 4D, these sequences favored hydrophobic substitutions, particularly alanine (A) and phenylalanine (F). Notably, the designed mutations showed site-specific preferences for charged residues rather than universal avoidance: Some positions exhibited a trend toward mutation to acidic or basic amino acids, while other positions disfavored charged side chains. This pattern is likely dictated by the electrostatic properties of the inner surface of the MHC-II binding pocket, where complementary or repulsive charge–charge interactions between the peptide epitope and pocket residues influence the formation of stable binding interfaces, a trend partially consistent with observations in the PMD variant series. To further quantify these differences, we generated sequence logos to visualize amino acid usage frequencies at mutation positions across PMD and PMI mutants (Fig. 4E and F). This analysis confirms that PMD and PMI exhibit distinct amino acid preference patterns: PMD mutants favor residues that disrupt MHC-II binding, whereas PMI mutants prefer residues that strengthen interactions within the binding groove.

Next, the top 10 sequences ranked by predicted antigen affinity changes were selected for structural similarity evaluation and further screening. The tetrameric structures of these mutant sequences were predicted using AF3, yielding average predicted local distance difference test (pLDDT) scores above 80, indicating high confidence in residue positioning. The predicted template modeling score (pTM) and interchain predicted TM score (ipTM) values were approximately 0.4 and 0.3, respectively, suggesting that AF3 is more confident in monomer predictions than in tetrameric assemblies (Fig. 5A to D). Comparison of scoring differences across predicted structures revealed that sequences, such as PMD-02, PMD-05, PMD-07, PMD-08, PMI-01, PMI-02, PMI-03, PMI-05, and PMI-09, tended to form higher-quality homomeric complexes in tetramer simulations. Subsequent structural alignment with the WT HBc tetramer (PDB ID: 6HTX) showed substantial variation in RMSD values among these sequences (Fig. 5E and F), indicating that both PMI and PMD series mutants exhibited relatively small deviations from the WT HBc VLP structure, with PMD-02, PMD-05, PMD-07, PMI-01, PMI-03, and PMI-09 performing the best.

**Fig. 5.**
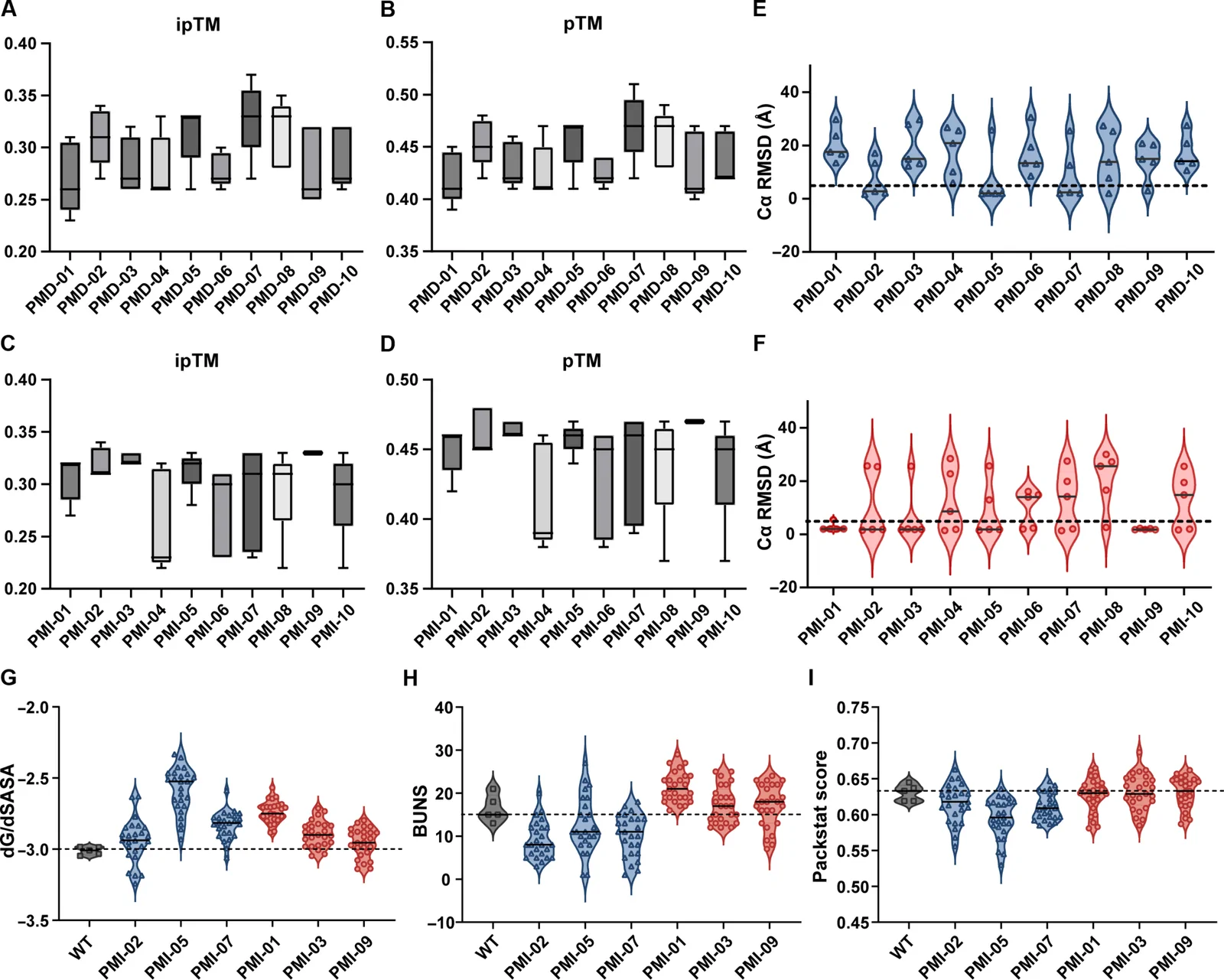
Predicted structural evaluation of ProteinMPNN-based deimmunization (PMD) and ProteinMPNN-based immunogenicity enhancement (PMI) mutant sequences. (A to D) Tetrameric structure predictions using AlphaFold3 (AF3). Predicted TM (pTM) scores of PMD (A) and PMI (B) mutant tetramers. Interchain predicted TM score (ipTM) scores of PMD (C) and PMI (D) mutant tetramers. pTM reflects the confidence in the global topology of the tetrameric assembly, while ipTM indicates confidence in interchain interactions. Values closer to 1 suggest high structural reliability. (E and F) Root mean square deviation (RMSD) values of PMD (E) and PMI (F) mutant tetramers aligned to the wild-type (WT) hepatitis B core (HBc) tetramer (Protein Data Bank [PDB] ID: 6HTX). Lower RMSD values indicate closer similarity to the WT HBc tetramer. (G to I) Interface analysis using Rosetta. (G) Gibbs free energy change per solvent-accessible surface area (dG/dSASA) reflecting the stability of tetramer interfaces (more negative values indicate stronger interactions). (H) Buried unsatisfied hydrogen bonds (BUNS), with lower values suggesting better interface packing. (I) Packstat score, with higher values indicating more compact and favorable packing.

We further analyzed the tetrameric interfaces using Rosetta, focusing on parameters such as dG/dSASA, BUNS, and Packstat. The dG/dSASA values reflect the propensity for dimer–dimer association; notably, PMD-05, despite low RMSD, exhibited high dG/dSASA values, indicating potential difficulty in forming stable tetramers. Packstat scores for these mutants were also relatively poor. In contrast, PMI-01 displayed favorable RMSD and Packstat values, comparable to or exceeding those of the WT HBc VLPs, although its dG/dSASA and BUNS scores were less optimal, which may impact overall stability (Fig. 5G to I). Overall, these results suggest that ProteinMPNN-based mutations have minimal impact on structural integrity, aligning with our design expectations and the observed amino acid preferences are consistent with rational design principles.

### Experimental validation of VLPIM-designed sequences

Based on the aforementioned sequence selection steps, 4 mutant sequences were chosen for experimental validation, including immunogenicity-reducing sequences PMD-02 and PMD-05 and immunogenicity-enhancing sequences PMI-01 and PMI-03. All sequences were synthesized and expressed in *E. coli*. Following the multicolumn purification strategies including size exclusion, anion exchange, and lipopolysaccharide–affinity chromatography, we successfully obtained expressed and purified products for all 4 sequences. SDS-PAGE analysis (Fig. S1) revealed that all 4 mutants were expressed at high levels and some of them achieved high purity (>90% by semiquantitative SDS-PAGE) and low endotoxin content (<0.5 EU/ml).

To evaluate whether these multisite mutants could assemble into VLP structures, we characterized the purified proteins by TEM (Fig. 6A). Mutants PMD-05 and PMI-03 failed to form VLP structures and existed primarily as monomers or small oligomers. This result suggests that the introduction of multiple mutations disrupted dimeric and tetrameric interactions, impairing proper self-assembly. In contrast, PMD-02 and PMI-01 successfully formed complete VLPs with morphology and particle size comparable to HBc VLPs, validating the feasibility of our computationally guided mutation and screening strategy. Importantly, these experimental outcomes are consistent with prior AF3 tetramer structure predictions, indicating that RMSD and related parameters can effectively guide large-scale sequence screening for structural integrity. We then analyzed the particle size of VLP-forming mutants based on TEM results. PMI-01 exhibited diameters similar to WT HBc VLPs, approximately 28 nm, indicating that the mutation did not disrupt VLP assembly. In contrast, PMD-02 showed a broader size distribution, around 50 nm (Fig. 6B to D). These findings imply that mutations in PMI-01 did not interfere with VLP assembly, likely because the mutation sites were outside the α5-helix domain.

**Fig. 6.**
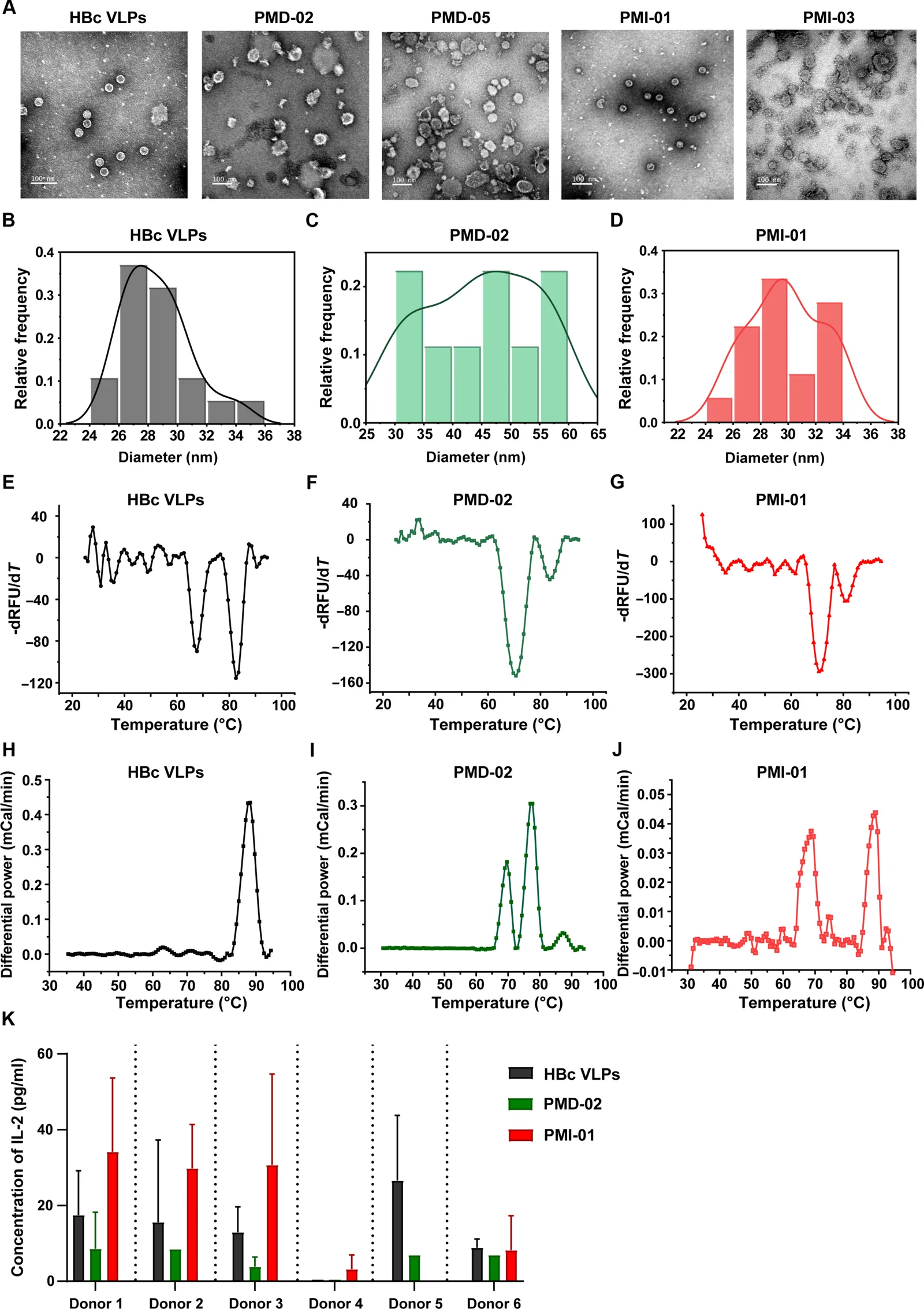
Characterization and immunogenic evaluation of Virus-Like Particle Immunogenicity Modulation (VLPIM)-designed hepatitis B core virus-like particle (HBc VLP) mutations. (A) Transmission electron microscopy (TEM) images of HBc VLPs, PMD-02, PMD-05, PMI-01, and PMI-03. PMD, ProteinMPNN-based deimmunization; PMI, ProteinMPNN-based immunogenicity enhancement. (B to D) Particle size distribution of HBc VLPs, PMD-02, and PMI-01. (E to G) DSF analysis of thermal stability for HBc VLPs, PMD-02, and PMI-01. *n* = 3. dRFU/dT stands for the first derivative of relative fluorescence unit (RFU) with respect to temperature. (H to J) Differential scanning calorimetry (DSC) analysis of thermal stability for HBc VLPs, PMD-02, and PMI-01. *n* = 1. (K) Changes in interleukin-2 (IL-2) levels in the supernatants of peripheral blood mononuclear cells (PBMCs) following stimulation with mutant HBc VLPs, including PMD-02 and PMI-01 (*n* = 6 donors, 4 replicates per donor).

In addition to morphological analysis, we assessed thermal stability differences using DSF and DSC. DSF results (Fig. 6E to G) displayed that PMD-02 and PMI-01 had 2 melting peaks similar to WT HBc VLPs, with Tm values around 70 and 83 °C, respectively, although the peak intensities varied, indicating that mutations slightly affected thermal stability. DSC measurements (Fig. 6H to J) provided higher sensitivity, revealing additional melting behaviors: HBc VLPs and PMI-01 had similar major Tm peaks around 88 °C. Minor peaks were observed at ~65 to 68 °C for PMI-01, reflecting slightly reduced stability due to multiple mutations. PMD-02 displayed 2 distinct peaks at 69.70 and 77.06 °C, indicating markedly decreased thermal stability. These results suggest that the peaks at ~88 °C correspond to the loss of VLP particle structure, while lower-temperature peaks represent partial disassembly of multimers or oligomers. Overall, PMI-01 showed slightly reduced thermal stability, whereas PMD-02 exhibited substantially lower stability compared to WT HBc VLPs.

To investigate the effect of multisite cooperative mutations on VLP immunogenicity, we incubated PMD-02 and PMI-01 with PBMCs from 6 healthy donors (final concentration, 10 μg/ml; 37 °C and 5% CO_2_). IL-2 secretion in the culture supernatant was measured by ELISA after 60 h. As shown in Fig. 6K, PMI-01 successfully activated IL-2 secretion in 5 of 6 donor samples (83.3%), with overall secretion levels higher than those observed for WT HBc VLPs or the other mutants. In the 3 donor PBMCs exhibiting strong responses, IL-2 concentrations after PMI-01 treatment were 34.05, 29.8, and 30.69 pg/ml, corresponding to 2.0-, 5.3-, and 2.4-fold increases relative to WT HBc VLPs, indicating that epitope optimization significantly enhanced cross-presentation via MHC-II molecules. In contrast, IL-2 secretion in PMD-02-treated PBMCs was lower than WT HBc VLPs. Notably, considering PMD-02 formed more uniform VLPs, the success rate of deep-learning-designed mutants (PMI/PMD series, 50%) was remarkably high, highlighting the potential of this strategy for large-scale sequence screening.

## Discussion

While deep-learning-based immunogenicity prediction tools have become increasingly powerful and widely adopted, their systematic use for controlled immunogenicity modulation, either attenuation or enhancement, remains insufficiently explored, particularly in complex multimeric systems such as VLPs. In this study, we present VLPIM, a computational framework designed to address the immunogenicity-related challenges of VLPs and to enhance their utility as nanocarriers and vaccine platforms. Our results highlight the potential of applying computational tools for protein design to multivalent nanostructures, while underscoring the importance of integrative frameworks that combine computational prediction with structural and biological validation.

This computational strategy consists of the following steps: (a) identification of dominant epitopes—experimentally validated data from the IEDB were integrated with NetMHCIIpan predictions to identify high-affinity MHC-II binding regions as mutation targets; (b) mutant library construction—targeted mutations were introduced into these hotspot regions using the deep learning model ProteinMPNN, generating variant libraries aimed at either reducing or enhancing immunogenicity; (c) virtual screening—ProteinMPNN-derived variants were reevaluated with NetMHCIIpan, and the top 10 candidates were structurally predicted using AF3. RMSD, pTM, ipTM, and Rosetta interface analyses were then applied to evaluate their capacity to assemble into VLPs. Importantly, adjustable parameters such as the mutation region and mutation number enable users to fine-tune the workflow for different VLP sequences, ensuring both flexibility and robustness in immunogenicity modulation while preserving structural integrity.

To validate the framework, we applied it to engineer HBc VLPs with altered immunogenicity. Extensive prior studies on HBc VLP engineering have demonstrated that epitope insertion at the N terminus, C terminus, or the major immunodominant region can enhance immunogenicity while preserving capsid assembly [46,47]. These approaches typically introduce exogenous epitopes into structurally tolerant regions. In contrast, the present study focuses on modulating endogenous immunogenicity through targeted sequence optimization within the native protein framework. In addition, we acknowledge that MHC-II binding affinity alone does not fully determine immunogenicity, as antigen processing and structural context also influence CD4^+^ T cell activation. In the context of VLPs, internalization and endosomal/lysosomal degradation enable peptide presentation on MHC-II, potentially exposing epitopes even from partially buried regions. Therefore, mutations were designed with consideration of overall structural integrity rather than being restricted to surface-exposed residues, allowing a balance between immunogenicity modulation and capsid assembly. According to the experimental results, PMI-01 successfully self-assembled into particles resembling WT HBc VLPs, while PMD-02 formed VLPs with broader and less uniform size distributions. Notably, PMD-02 exhibits reduced thermal stability compared with the WT HBc VLPs. Because reduced structural stability would be expected to facilitate rather than impede endosomal/lysosomal degradation, the concurrent reduction in IL-2 secretion supports the interpretation that the observed immunogenicity differences are primarily attributable to altered CD4^+^ T cell epitope recognition rather than to secondary effects on antigen processing efficiency. These results demonstrate that rational design combined with deep learning can generate VLP variants capable of modulating immunogenicity while retaining assembly competence. Although experimental validation was performed using the HBc VLP scaffold, VLPIM was designed as a scaffold-agnostic pipeline whose core components rely on general biophysical principles rather than scaffold-specific parameters, requiring no retraining for application to alternative systems. Scaffolds with lower intrinsic stability or distinct symmetry architectures may exhibit a narrower mutation-tolerant design space, necessitating more stringent energy thresholds or iterative validation cycles to ensure proper assembly and functional integrity.

Despite these promising results, several limitations remain. First, while NetMHCIIpan represents the current state of the art in MHC-II epitope prediction and effectively integrates experimental binding data, its predictive performance still has inherent limitations, partly due to constraints in available experimental epitope datasets. This introduces uncertainty in the initial epitope selection and ranking process. In addition, limited by the number of experimentally validated variants in this study, a rigorous assessment of sensitivity to initial epitope selection is not yet feasible. Nevertheless, the consistency between computational predictions and experimental immunogenicity trends supports the epitope ranking strategy as a reasonable basis for candidate selection, establishing proof of concept for the VLPIM framework.

Second, structural prediction also encounters difficulties. AF3 showed suboptimal performance in predicting multimeric assemblies such as VLPs, a limitation that has been reported by others as well [31]. The structural modeling component of the VLPIM workflow, combining AF3 prediction, RMSD evaluation, and Rosetta-based energetic analysis, serves as a structure-informed prioritization step rather than a deterministic predictor of assembly competence. Among the 4 top-ranked variants subjected to experimental validation, 2 successfully assembled into intact VLPs, consistent with moderate filtering capacity. Quantitative assessment of the relationship between computational metrics and assembly outcomes will require larger experimentally validated datasets and this will remain an important direction for future development of the framework. In future iterations of the framework, incorporating experimental structural constraints such as cryo-electron-microscopy density maps or small-angle x-ray scattering-derived distance distributions could improve the accuracy of multimeric assembly predictions. Such hybrid computational–experimental approaches represent a promising direction for addressing current limitations in modeling higher-order VLP architectures.

In addition, challenges remain in experimental validation. Current in vitro and in vivo immunogenicity assays are not well integrated, complicating their use in high-throughput screening and hindering the feedback loop between computational design and experimental testing. Although IL-2 secretion from PBMC cultures provides a sensitive and widely used indicator of early CD4^+^ T cell activation, it represents only a partial view of the overall T cell response. The assay primarily captures early activation signals and does not distinguish between different functional T cell subsets, such as effector versus regulatory populations, nor does it resolve T helper polarization (e.g., Th1 versus Th2 responses). In addition, IL-2 production alone does not directly demonstrate MHC-II-restricted antigen presentation. Therefore, while the observed differences in IL-2 secretion among structural variants support differential modulation of T cell activation potential, they do not fully define the functional phenotype or mechanistic basis of the responding T cell populations.

Another limitation relates to donor variability in PBMC responses. To partially address this, PBMCs from 6 independent donors were tested across all experimental groups in a paired design, enabling relative comparisons within the same donor-specific immune context. However, HLA typing was not performed for the PBMC samples used here, limiting interpretation of allele-specific effects. Future studies with larger donor cohorts and defined HLA genotypes would enable stratified analysis of responders versus nonresponders and more detailed investigation of allele-specific immune responses.

An important limitation of the current study is the exclusive focus on cellular immune readouts. The intrinsic antigenicity of VLPs is largely dictated by their pathogen-associated molecular patterns, which efficiently drive B cell activation and are therefore difficult to eliminate through sequence-level modifications. In this context, modulation of CD4^+^ T cell epitopes may indirectly influence B cell responses and antibody cross-reactivity by altering the availability of cognate CD4^+^ T cell help for B cells. However, due to fundamental interspecies differences in MHC-II repertoires, conventional mouse models are not well suited for further evaluation of humoral immune responses, which substantially increases experimental complexity and limits the direct assessment of humoral immune outcomes.

Future studies incorporating antigen-specific assays, broader cytokine profiling, phenotypic characterization of T cell subsets, proliferation assays, and MHC-II blocking experiments will be necessary to provide in-depth mechanistic insight.

VLPIM serves as an in silico tool that follows rational design principles, accelerating and lowering the cost of the design process. Nevertheless, future improvements could further enhance its capabilities. For example, inspired by the success of solMPNN [48] (a fine-tuned version of ProteinMPNN) and advances in antibody humanization, integrating reorganized protein sequence–structure databases could enable deeper exploitation of existing folding models. This would allow more comprehensive and precise modulation of immunogenicity. Furthermore, while our current workflow focuses primarily on accelerating mutation screening, it does not fully leverage available biological data. Incorporating large-scale human protein sequence and structural information could refine ProteinMPNN to better capture sequence design rules, thereby improving the efficiency and accuracy of the design process. Similarly, NetMHCIIpan and AF3 could be integrated more directly into iterative filtering and optimization cycles.

## Conclusion

In conclusion, we developed an integrated framework (VLPIM) for rationally modulating the immunogenicity of VLPs while preserving structural integrity. The pipeline begins by identifying dominant CD4^+^ T cell epitopes using NetMHCIIpan alongside experimentally validated data, followed by targeted mutation generation with ProteinMPNN. Top candidates were screened for immunogenicity via NetMHCIIpan and subsequently analyzed for structural fidelity using AF3 and Rosetta. Applying this framework to HBc VLPs successfully yielded mutants with precisely enhanced (PMI-01) or reduced (PMD-02) immunogenicity, while maintaining overall particle assembly. Together, these findings establish a robust platform for tunable VLP design that integrates artificial-intelligence-driven protein engineering with structural and immunological validation.

## Data Availability

The standalone version of VLPIM has been made available on GitHub (https://github.com/RuijinHospitalVNAR/VLPIM) and is freely available for noncommercial users. The VLPIM web server is accessible at https://ruijinhospitalvnar.github.io/Toolboxes/VLPIM_Web_services/.
