# Supplementary material for: Rational Immunogenicity Modulation of Virus-Like Particles by VLPIM
Source: Comput Struct Biotechnol J. 2026 Apr 28;35(3):0063. doi: 10.34133/csbj.0063 (PMC13632935; doi:10.34133/csbj.0063)
Supplement: Supplementary file 1 [file csbj.0063.f1.docx]

**Supporting information**

**Rational Immunogenicity Modulation of Virus-like Particles by VLPIM**

**Figure S1**. SDS-PAGE analysis of PMD-02, PMD-05, PMI-01 and PMI-03.

**Table S1.** Top 20 ProteinMPNN-generated variants ranked by NetMHCIIpan-predicted changes in immunogenicity.

| **Variant ID** | **Sequence** |
| --- | --- |
| **HBc VLPs** | MDIDPYKEFGATVELLSFLPSDFFPSVRDLLDTASALYREALESPEHCSPHHTALRQAILCWGELMTLATWVGVNLEDPASRDLVVSYVNTNMGLKFRQLLWFHISCLTFGRETVIEYLVSFGVWIRTPPAYRPPNAPILSTL |
| **PMD-01** | MDIDPYKEFGATVELMSNLPDDYFPSVDELLDTASALYREALESPEHCSPHHTALRQAILCWGELMTLATWVGVNLEDPASRDLVVSHVADTLGKRYRQLLWFHISCLTFGRETVIEYLVSFGVWIRTPPAYRPPNAPILSTL |
| **PMD-02** | MDIDPYKEFGATVELMSNLPDDYFPSVEELLDTASALYREALESPEHCSPHHTALRQAILCWGELMTLATWVGVNLEDPASRDLVVNYVENELGLRYRQLLWFHISCLTFGRETVIEYLVSFGVWIRTPPAYRPPNAPILSTL |
| **PMD-03** | MDIDPYKEFGATVELMSNLPDDYFPSVEELLDTASALYREALESPEHCSPHHTALRQAILCWGELMTLATWVGVNLEDPASRDLVVNYVNETLGKRYRQLLWFHISCLTFGRETVIEYLVSFGVWIRTPPAYRPPNAPILSTL |
| **PMD-04** | MDIDPYKEFGATVELMSNLPDDYFPSVDELLDTASALYREALESPEHCSPHHTALRQAILCWGELMTLATWVGVNLEDPASRDLVVTHVTTTLGKRYRQLLWFHISCLTFGRETVIEYLVSFGVWIRTPPAYRPPNAPILSTL |
| **PMD-05** | MDIDPYKEFGATVELMSNLPDDYYPSVEELLDTASALYREALESPEHCSPHHTALRQAILCWGELMTLATWVGVNLEDPASRDLVVTRVHTELGRRYRQLLWFHISCLTFGRETVIEYLVSFGVWIRTPPAYRPPNAPILSTL |
| **PMD-06** | MDIDPYKEFGATVELMSNLPEDYFPSVEELLDTASALYREALESPEHCSPHHTALRQAILCWGELMTLATWVGVNLEDPASRDLVVTRVREELGKRYRQLLWFHISCLTFGRETVIEYLVSFGVWIRTPPAYRPPNAPILSTL |
| **PMD-07** | MDIDPYKEFGATVELMSNLPDDYFPSVEELLDTASALYREALESPEHCSPHHTALRQAILCWGELMTLATWVGVNLEDPASRDLVVNYVNDTLGVRYRQLLWFHISCLTFGRETVIEYLVSFGVWIRTPPAYRPPNAPILSTL |
| **PMD-08** | MDIDPYKEFGATVELMSNLPDDYFPSVEELLDTASALYREALESPEHCSPHHTALRQAILCWGELMTLATWVGVNLEDPASRDLVVNYVKETLGIRYRQLLWFHISCLTFGRETVIEYLVSFGVWIRTPPAYRPPNAPILSTL |
| **PMD-09** | MDIDPYKEFGATVELMSNLPDDYFPSVEELLDTASALYREALESPEHCSPHHTALRQAILCWGELMTLATWVGVNLEDPASRDLVVNYVTTELGKRYRQLLWFHISCLTFGRETVIEYLVSFGVWIRTPPAYRPPNAPILSTL |
| **PMD-10** | MDIDPYKEFGATVELMSQLPDDYFPSVEELLDTASALYREALESPEHCSPHHTALRQAILCWGELMTLATWVGVNLEDPASRDLVVTHVRETLGRHYRQLLWFHISCLTFGRETVIEYLVSFGVWIRTPPAYRPPNAPILSTL |
| **PMI-01** | MEIDPFRAFGSSLELMSFLPSDFFPSVRDLLDTASALYREQLRSAEHCSPEITALRQAILCWGELMTLATWVGVNLEDPASRDLVVSYVNTNMGLKFRQLLWFHISCLTFGRETVIEYLVSFGVWIRTPPAYRPPNAPILSTL |
| **PMI-02** | MKIDPFRAFGSSEELMSFLPSDFFPSVRDLLDTASALYREELRSARHCSPEITALRQAILCWGELMTLATWVGVNLEDPASRDLVVSYVNTNMGLKFRQLLWFHISCLTFGRETVIEYLVSFGVWIRTPPAYRPPNAPILSTL |
| **PMI-03** | MQIDPFRAFGSSIELMSFLPSDFFPSVRDLLDTASALYRAQLQSSEHCSPEITALRQAILCWGELMTLATWVGVNLEDPASRDLVVSYVNTNMGLKFRQLLWFHISCLTFGRETVIEYLVSFGVWIRTPPAYRPPNAPILSTL |
| **PMI-04** | MKIDPFRAFGSSLELMSFLPSDFFPSVRDLLDTASALYREQLQSDQHCSPEITALRQAILCWGELMTLATWVGVNLEDPASRDLVVSYVNTNMGLKFRQLLWFHISCLTFGRETVIEYLVSFGVWIRTPPAYRPPNAPILSTL |
| **PMI-05** | MKIDPFRAFGSSEELMSFLPSDFFPSVRDLLDTASALYREELQSAKHCSPEITALRQAILCWGELMTLATWVGVNLEDPASRDLVVSYVNTNMGLKFRQLLWFHISCLTFGRETVIEYLVSFGVWIRTPPAYRPPNAPILSTL |
| **PMI-06** | MKIDPFRPFGSSLELMSFLPSDFFPSVRDLLDTASALYREELRSPRHCSPEITALRQAILCWGELMTLATWVGVNLEDPASRDLVVSYVNTNMGLKFRQLLWFHISCLTFGRETVIEYLVSFGVWIRTPPAYRPPNAPILSTL |
| **PMI-07** | MRIDPFRAFGSSEELMSFLPSDFFPSVRDLLDTASALYREQLQSDRHCSPEITALRQAILCWGELMTLATWVGVNLEDPASRDLVVSYVNTNMGLKFRQLLWFHISCLTFGRETVIEYLVSFGVWIRTPPAYRPPNAPILSTL |
| **PMI-08** | MRIDPFRAFGSSEELMSFLPSDFFPSVRDLLDTASALYREQLQSDRHCSPEITALRQAILCWGELMTLATWVGVNLEDPASRDLVVSYVNTNMGLKFRQLLWFHISCLTFGRETVIEYLVSFGVWIRTPPAYRPPNAPILSTL |
| **PMI-09** | MKIDPFRAFGSSLELMSFLPSDFFPSVRDLLDTASALYREELQSPKHCSPHHTALRQAILCWGELMTLATWVGVNLEDPASRDLVVSYVNTNMGLKFRQLLWFHISCLTFGRETVIEYLVSFGVWIRTPPAYRPPNAPILSTL |
| **PMI-10** | MNVDPFRAFGSSEELMSFLPSDFFPSVRDLLDTASALYRAELQSAKHCSPFHTALRQAILCWGELMTLATWVGVNLEDPASRDLVVSYVNTNMGLKFRQLLWFHISCLTFGRETVIEYLVSFGVWIRTPPAYRPPNAPILSTL |

Variants are listed from top to bottom by their predicted immunogenicity shift. Residues in deimmunization target regions are shown in blue font; residues in immunogenicity enhancement target regions are shown in red font. All mutation sites are highlighted in yellow.

**Figure S2**. Mapping of predicted MHC-II epitopes onto the 3D structure of the VLP. PMI regions are colored in red and PMD regions are colored in blue.
